# A longitudinal study on *Anopheles *mosquito larval abundance in distinct geographical and environmental settings in western Kenya

**DOI:** 10.1186/1475-2875-10-81

**Published:** 2011-04-10

**Authors:** Susan S Imbahale, Krijn P Paaijmans, Wolfgang R Mukabana, Ron van Lammeren, Andrew K Githeko, Willem Takken

**Affiliations:** 1 Laboratory of Entomology, Wageningen University and Research Centre, P.O. Box 8031, 6700 EH Wageningen, The Netherlands; 2 Kenya Medical Research Institute, Centre for Global Health Research, P.O Box 1578, 40100, Kisumu, Kenya; 3 Center for Infectious Disease Dynamics and Department of Entomology, Pennsylvania State University, University Park, PA16802, USA; 4 School of Biological Sciences, University of Nairobi, P.O. Box 30197-00100 GPO, Nairobi, Kenya; 5 International Centre of Insect Physiology and Ecology, P.O. Box 30772 - 00100 GPO, Nairobi, Kenya; 6 Laboratory of Geo-information Science and Remote Sensing, Wageningen University and Research Centre, P.O Box 47, 6700AA, Wageningen, The Netherlands

## Abstract

**Background:**

As the ecology of mosquito larvae can be complex there is need to develop a rational framework for undertaking larval ecological studies. Local environmental characteristics, such as altitude, climate and land use, can significantly impact on phenology and population dynamics of mosquito larvae, and indirectly affect the dynamics of mosquito-borne diseases. The aim of this study was to assess the feasibility of implementing an integrated approach to larval source management under the distinct ecological settings.

**Methods:**

The study was conducted in two highland villages and one village, at a lower altitude, in the Lake Victoria basin, where malaria is endemic and transmitted by the same *Anopheles *mosquito species. In each village the stability of mosquito larval habitats was classified as either temporary or permanent. The productivity of these habitat types was quantified by carrying out weekly larval sampling using a standard dipping method for a period of two years. During sampling the physical characteristic of the larval habitat, including the vegetation cover were noted. Ambient temperature, rainfall and relative humidity were recorded on a 21 × Micro-datalogger in each study site.

**Results:**

*Anopheles gambiae *sensu lato larvae were found in all study sites. *Anopheles arabiensis *was more abundant (93%) in Nyalenda (Lake Victoria basin) and Fort Ternan (highland area; 71%). In Lunyerere (highland area), *An. gambiae sensu stricto *comprised 93% of the total *An. gambiae *s.l. larvae. Larvae of *An. gambiae *s.l. mosquitoes were present in both temporary and permanent habitats with monthly variations dependent on rainfall intensity and location. *Anopheles *larvae were more likely to be found in man-made as opposed to natural habitats. Grassy habitats were preferred and were, therefore, more productive of *Anopheles *larvae compared to other habitat types. Weekly rainfall intensity led to an increase or decrease in mosquito larval abundance depending on the location.

**Conclusion:**

The majority of mosquito breeding habitats were man made in all sites. Both temporary and permanent habitats were suitable for *An. gambiae *breeding. In Fort Ternan temporary sites were favoured for mosquito breeding above permanent sites. Significant differences in larval abundance were found depending on weekly rainfall intensity. Larval source management programmes should target permanent and temporary habitats equally and work closely with land and home owners as a majority of the breeding habitats are man made.

## Background

In western Kenya, the sibling mosquito species *Anopheles arabiensis *and *Anopheles gambiae sensu stricto *(hereafter referred to as *An. gambiae*), and *Anopheles funestus*, are the principal vectors of malaria. Although *An. gambiae *is usually the predominant species in environments with high humidity and rainfall, *An. arabiensis *is more common in zones with less rainfall and both species occur sympatrically across a wide range of tropical Africa [1,2]. Larvae of *An. gambiae *are commonly found in clear, sunlit pools of water in small depressions such as foot or hoof prints, the edges of bore holes and burrow pits, roadside puddles formed by tire tracks, irrigation ditches and other man-made shallow water bodies [3-7]. *Anopheles gambiae *malaria vectors have also been found breeding in polluted water rich in organic matter [8-11], in large bodies of water such as flood plains [12] and in pools of water along lake shores especially when there are fluctuations in water level as it occurs in Lake Victoria [13]. Environmental alterations due to deforestation, swamp reclamation mainly for agriculture, excavation of sand and building stones, brick making and vegetation clearance may lead to an increase in larval habitats of malaria vectors, such as *An. gambiae *[14-18].

Agricultural areas represent moist, disturbed environments that can sustain both immature and adult mosquitoes [19]. Water storage and irrigation are often necessary to sustain agricultural activity within the urban context. Therefore, urban farming may be increasing the availability of potential habitats for *Anopheles *mosquitoes [8,11]. The elimination of papyrus swamps in the Ugandan highlands created conducive breeding habitats for *An. gambiae *and *An. funestus*, leading to increased malaria transmission [20]. In the highlands of western Kenya, *An. gambiae *emerged from habitats with agricultural crops only on land that had been cleared from forests and swamps [21]. The successful breeding of anopheline mosquitoes was thought to be caused by the higher temperatures in the cleared habitats compared to the forest. Likewise, the absence of a suitable habitat and increased water pollution generally inhibits the development of anopheline larvae in urban centers, resulting in fewer *Anopheles *mosquitoes [22,23].

Knowledge on anopheline larval ecology is spatially limited and often insufficient to achieve effective vector control through means of larval control [24,15]. From existing literature, little is known about the habitats, abundance and distribution of the larvae of the main African malaria vectors. Systematic research on the larval ecology of the main malaria vectors in Africa is still limited and often represents short periods (less than 12 months) of data collection, frequently considering mosquito-infested habitats only [15]. The main objective of the present study was to find out the suitability, stability and productivity of habitats that served as potential anopheline breeding habitats, with emphasis on malaria vector larval habitats in two highland villages and one peri-urban area, at lower altitude, in western Kenya, where malaria is endemic with large seasonal and geographic variations. The results provide baseline data essential for finding suitable and effective larval source management strategies for each of the studied areas.

## Methods

### Study area

The study was undertaken in two highland villages (Fort Ternan and Lunyerere) and a peri-urban area (Nyalenda) in western Kenya from March 2006 to March 2008. Fort Ternan and Lunyerere are at a similar altitude but they differ in topography, vegetation cover and land use, while Nyalenda, situated in the Lake Victoria basin, was chosen for comparison. Lunyerere and Fort Ternan lie 73.14 km apart and 24.81 km from Kisumu City. Western Kenya has a bimodal pattern of rainfall with long rains occurring from April to June and short rains between November and December with yearly variability.

Fort Ternan (0°12'South and 35°20'East) is a rural village, with approximately 2,000 inhabitants. It lies within the Kericho County located on the slopes of the Nandi hills, between an altitude of 1,500-1,650 m above sea level. The area is hilly with V-shaped valleys that drain water into the Kipchorian River. Typical for this area is the traditional practice of large scale farming of cash (sugarcane) and food (maize, vegetables) crops in addition to livestock keeping. Several coffee and tea farms are also within the area. In recent years the Fort Ternan area has not undergone many land use changes.

Lunyerere (0°06'North and 34°43'East) is a small village with approximately 4,000 inhabitants. The village is located in Vihiga County, on the western side of Kakamega Forest, about 5 km north of the equator. The area under study has an altitude ranging from 1,520-1,560 m. The district is hilly and cold, characterized by undulating hills that often form basin-shaped valleys that are prone to flooding, offering excellent mosquito breeding habitats. Farming for both cash (tea) and food crops (maize, vegetables) is the main economic activity. Lunyerere was, until recently, characterized by highland rainforest and natural swamps (mostly papyrus) in the valley floor, which have been transformed into crop-land in the past 20 years. Characteristic to this area is the underground seepage of water that is always present, offering good breeding habitats for mosquitoes the whole year.

Nyalenda (0°06'South and 34°46' East, 1250-1280 m altitude) is a low-income peri-urban area located on the eastern outskirts of Kisumu city, with approximately 26,000 inhabitants. Kisumu is situated in the Victoria Lake basin. This area is hot and humid, and highly endemic for malaria. The study area is flat, and fed by natural springs that produce abundant quantities of water used for irrigation of small scale gardens and commercial tree nurseries. About 10 years ago, nearly 70% of Nyalenda was a natural swamp; however, the expansion of Kisumu town led to development activities in the area. The area is occupied by a low-income population and due to population pressure and the need for food, the original swamplands have been transformed into irrigated rice, vegetable and horticultural farms. The area has unplanned sewerage facilities, so raw sewage often floods the swamp.

## Environmental factors

### Larval habitat characterization

A preliminary survey of suitable anopheline mosquito breeding habitats was done in March 2006, by searching for areas where water stagnates in the three study sites. Suitability was determined by the presence of immature stages of mosquitoes. The bodies of water identified were then classified according to their nature: river fringes (habitats formed along river banks when the water level drops), rain pools, drainage channels, erosion pits (deep holes formed when water flows down hill), artificial watering points (leaking taps and damaged pipes were grouped together), hoof-prints, tire tracks, rice paddies and burrow pits (holes made by or for animals). Vegetation was broadly grouped into five categories namely algae, grass, papyrus reeds, agricultural crops (crops used by the farmers either as food, such as arrowroot, rice and vegetables or as fodder, such as Napier grass) and vegetation types not included in the other categories. Habitats without any vegetation were grouped under 'no vegetation'. Vegetation was measured visually by estimating the percentage of the larval habitat covered. In case of mixed vegetation, the dominant vegetation (covering >50%) in the habitat was recorded. The larval habitats were finally grouped according to their stability into temporary habitats and permanent habitats. Temporary habitats held water for a short period of time (until approximately two weeks after the rainy season had ended) and stem mainly from rain showers; when rain ceased these habitats dried out. The permanent sites, on the other hand, held water for a longer period of time (approximately 2-3 months after the rains ended or fed by natural underground sources) and hence were more stable. In each site 10 temporary and 10 permanent habitats were selected for mosquito larval sampling. The permanent habitats remained in the same location throughout the sampling period while temporary habitats changed depending on the availability of water. To determine productivity, the habitats were examined once per week for the presence of aquatic stages of anopheline and culicine mosquitoes. In addition, the perimeter of the water surface was recorded as ≤ 10 m, 10-100 m and >100 m.

### Meteorological data

Automated weather stations were installed, one at Fort Ternan Health centre (1550 m altitude), one at Lyanaginga Health Centre (1500 m altitude) about 30 km from Lunyerere and a third station at the Kenya Medical Research Institute (KEMRI), Centre for Global Health Research, Kisian (1100 m altitude) about 17 km from Nyalenda. The weather stations measured temperature and humidity at 2 m above ground (ventilated probe; Vaisala, Finland) and precipitation (rain gauge, Eijkelkamp, The Netherlands) throughout the study period. The weather variables were recorded on a 21 × Micro-datalogger (Campbell Scientific Inc., UK) at an interval of 15 minutes from March 2006 to March 2008. Technical problems were experienced with the weather station installed at KEMRI from May to November 2006 and from June to October 2007. For this period the data presented were obtained from the Kenya Airports Authority, Kisumu airport 10 km away from KEMRI. Relative humidity (between May to November 2006 and June to October 2007) is reported as the mean of two values collected in the morning (06.00 hours) and noon (12.00 hours). In the highland sites during the month of November 2007, the rain gauges experienced technical problems and hence the data for this period are excluded from the analysis.

## Ecology

### Larval sampling

Weekly surveys were conducted to establish the availability of stagnant water, habitat characteristics and larval densities from March 2006 to March 2008 in all habitats identified. Larval sampling was done using the standard dipping method with a 350 ml mosquito scoop (Bioquip, Gardena, CA, USA) as described by Service [25]. The number of dips taken from each habitat was dependent on the perimeter of the larval habitat. Larval habitats were categorized depending on their size in perimeter. The perimeter was grouped into three classes as ≤ 10 m, 10-100 m and >100 m, and a maximum of 10, 50 and 150 dips were taken from these habitats, respectively. The number of larvae from each habitat was then expressed as the larvae per dip (total number of larvae/no of dips). Both anopheline and culicine larvae were sampled and the stage of larval development recorded as either 1^st^-2^nd ^instars (early), 3^rd^-4^th ^(late) instars or pupae. A portion of the late instars were immediately preserved in 90% absolute ethanol and taken to the laboratory at the Kenya Medical Research Institute (KEMRI), Kisumu for identification under a compound microscope according to the keys of Gillies and Coetzee [26]. Microscopically identified *An. gambiae *late instars were preserved individually in Eppendorf tubes containing absolute ethanol pending further identification by polymerase chain reaction (PCR) [27].

## Ethical considerations

Institutional ethical clearance was given by the Kenya Medical Research Institute (KEMRI) and Wageningen University and Research Centre (WUR), The Netherlands, protocol approval number 1121 and 512. In addition permission was obtained from the community elders.

### Data analysis

General linear model (GLM) multivariate analysis of variance was used to calculate the estimated marginal means and 95% confidence intervals (C.I) in larval densities within the different habitat types and habitats with different vegetation cover. Generalized linear model, univariate analysis with a normal probability distribution and log-linked function was used in the calculation of the odds ratio (OR) and 95% Wald Confidence Intervals. Mosquito larval abundance in different habitat types was compared to that of rain pools while habitats with different plant cover types were compared to those without cover. Rain pools were chosen for comparison because they are (1) among the most preferred habitat type for *Anopheles *malaria vectors, (2) present in all the studied sites and (3) they occurred naturally depending on the topography of the area under study, whereas the rest of the habitat types were either man-made or formed as a result of human activities such as transport, farming and animal husbandry. Relative humidity and temperature data were averaged and rainfall data were pooled for each month. Cross-correlation analysis with a time lag of up to 7 weeks was then used to address the correlation of weekly larval densities with weekly rainfall. To find out whether rainfall intensity had any impacts on *Anopheles *larval abundance, the total weekly rainfall was grouped into four classes (1) none = 0 mm (2) low = 1-50 mm, (3) average = 51-100 mm and (4) high = 101-200 mm and then compared with larval abundance using univariate analysis.

## Results

### Larval habitat types and immature *Anopheles *abundance

In general, hoof prints, rain pools, tire tracks, containers and paddies were temporary in nature while drainage canals, erosion pits, river fringes, burrow pits and livestock watering points were permanent (Table 1). Drainage canals, hoof prints and rain pools were present in all study sites. Erosion pits were present in the highland villages, whereas paddies and burrow pits were only found in Nyalenda. In the highland villages of Fort Ternan and Lunyerere, the most frequently encountered larval habitats were drainage canals (89.2%) and livestock watering points (mostly piped water from taps) (25.9%). In Nyalenda, drainage canals and rice paddies represented 56.1% and 38.5% of all available larval sites, respectively (Table 1). Eighty six percent of all habitats recorded for the entire study period were as a result of human activities.

**Table 1:** The abundance of early and late anopheline larvae in different habitat types in Fort Ternan, Lunyerere and Nyalenda during the two years of weekly sampling

Site name	Habitat type	Habitat stability	Weekly sampling (%)	Early instars			Late instars		
				(EMM ± SE)	OR	P	(EMM ± SE)	OR	P
Fort Ternan	Rain pools	Temporary	4.4	0.060 ± 0.019	1		0.037 ± 0.009	1	
	Drainage canal	Permanent	14	0.036 ± 0.012	0.666	0.054**	0.036 ± 0.006	0.976	0.918
	Erosion pit	Permanent	17.7	0.102 ± 0.009	1.390	0.090	0.040 ± 0.004	0.913	0.676
	Hoof print	Temporary	13.6	0.131 ± 0.013	1.991	0.001*	0.069 ± 0.006	1.753	0.007*
	River fringe	Permanent	11.1	0.060 ± 0.019	0.641	0.052**	0.024 ± 0.006	0.412	0.002*
	Taps	Permanent	25.9	0.043 ± 0.010	0.431	0.000*	0.020 ± 0.005	0.325	0.000*
	Tire track	Temporary	13.4	0.052 ± 0.013	0.896	0.690	0.028 ± 0.006	0.797	0.327

Lunyerere	Rain pools	Temporary	1.5	0.124 ± 0.056	1		0.035 ± 0.023	1	
	Drainage canal	Permanent	89.2	0.167 ± 0.007	1.134	0.648	0.062 ± 0.003	1.249	0.535
	Erosion pit	Permanent	6.7	0.129 ± 0.027	1.019	0.956	0.046 ± 0.011	1.214	0.635
	Hoof print	Temporary	2.5	0.014 ± 0.044	0.234	0.069	0.006 ± 0.018	0.133	0.022*
	Container	Temporary	0.2	0.020 ± 0.176	1.000	-	0.012 ± 0.072	1.000	-

Nyalenda	Rain pools	Temporary	2.3	0.058 ± 0.033	1		0.002 ± 0.007	1	
	Drainage canal	Permanent	56.1	0.076 ± 0.11	1.624	0.255	0.014 ± 0.002	2.118	0.195
	Hoof print	Temporary	1.1	0.042 ± 0.047	0.692	0.488	0.000 ± 0.010	0.446	0.312
	Rice paddy	Temporary	39.5	0.135 ± 0.013	2.586	0.026*	0.014 ± 0.003	3.164	0.048*
	Burrow pit	Permanent	1.0	0.366 ± 0.48	7.302	0.003*	0.038 ± 0.010	7.781	0.010*

*Significantly different from rain pools **marginally significant from rain pools at P = 0.05, OR = Odds ratio; P = level of significance; EMM = estimated marginal means; SE = standard error of the mean

In Fort Ternan, the abundance of early instars reduced by over 30% (OR 0.666; P 0.054) in drainage canals and habitats along the river fringe (OR 0.641; P 0.052) in comparison to rain pools. Compared to rain pools, the river fringe contained 58% fewer (OR 0.412; P 0.002) late instars and the cattle watering points 67.5% fewer (OR 0.325; P < 0.001) late instars. In Lunyerere, the abundance of early and late instars was not significantly different (P > 0.05) among the habitat types except for late instars in hoof prints (P = 0.022) when compared to rain pools. There were 76.6% (OR 0.234; P >0.05) fewer first instars and 86.7% (OR 0.133; P >0.001) fewer late instars in hoof prints compared to rain pools. In Nyalenda, early instars were almost three times (OR 2.586; P < 0.05) and seven times (OR 7.302; P < 0.05) more abundant in rice paddies and burrow pits, respectively, compared to rain pools. Similar results were obtained for the abundance of late instars in paddies (OR 3.164; P < 0.05) and burrow pits (OR 7.781; P = 0.01). In hoof prints the abundance of early and late instars was 30% and 55% less, respectively, compared to that of rain pools (Table 1).

### Vegetation cover and immature Anopheles occurrence

Habitats that had grass, algae, other vegetation, as well as those without vegetation were present in different frequencies among the sites (Table 2). Papyrus reeds (swamp) and agricultural vegetation were present in Lunyerere and urban Nyalenda but not in Fort Ternan. The majority of larval habitats sampled in Fort Ternan (66.5%), Lunyerere (66.4%) and (62.8%) Nyalenda, had grass growing in them.

**Table 2:** The abundance of early and late anopheline larvae within habitats covered with different vegetation types in Fort Ternan, Lunyerere and Nyalenda during the 2 years of weekly sampling

Site	Dominant vegetation cover	Weekly sampling (%)	Early instars (EMM ± SE)	OR	P	Late instars (EMM ± SE)	OR	P
Fort Ternan	None	5.8	0.152 ± 0.047	1		0.096 ± 0.044	1	
	Algae	1.3	0.417 ± 0.097	2.181	0.014	0.292 ± 0.091	1.622	0.297*
	Grass	66.5	0.369 ± 0.014	2.075	< 0.001	0.304 ± 0.013	2.500	< 0.001
	Others	26.4	0.433 ± 0.022	2.432	< 0.001	0.306 ± 0.021	2.401	< 0.001

Lunyerere	None	9.5	0.096 ± 0.035	1		0.073 ± 0.034	1	
	Algae	3.6	0.233 ± 0.056	2.184	0.008	0.161 ± 0.055	1.985	0.060*
	Agricultural crops	9.5	0.354 ± 0.035	3.214	< 0.001	0.321 ± 0.035	3.861	< 0.001
	Grass	66.4	0.486 ± 0.013	4.369	< 0.001	0.452 ± 0.013	5.436	< 0.001
	Others	0.2	0.683 ± 0.272	6.378	< 0.001	0.346 ± 0.268	3.813	0.142*
	Papyrus reeds	10.9	0.222 ± 0.032	2.018	0.003	0.196 ± 0.032	2.359	0.002

Nyalenda	None	6.5	0.313 ± 0.044	1		0.241 ± 0.040	1	
	Algae	3.5	0.610 ± 0.059	2.056	< 0.001	0.350 ± 0.056	1.318	0.208*
	Agricultural crops	3.4	0.544 ± 0.060	1.847	0.001	0.270 ± 0.057	1.038	0.889*
	Grass	62.8	0.614 ± 0.014	2.076	< 0.001	0.326 ± 0.013	1.214	0.207*
	Others	18.4	0.517 ± 0.026	1.746	< 0.001	0.314 ± 0.024	1.159	0.379*
	Papyrus reeds	5.4	0.739 ± 0.048	2.507	< 0.001	0.249 ± 0.046	0.889	0.620*

*No significant difference with plots without vegetation cover. OR = Odds ratio; P = Level of significance; EMM = estimated marginal means; SE = standard error of the mean

Occurrence of larvae in habitats with different vegetation cover varied depending on the study area. However, early instars were distributed differently among the habitats with different vegetation cover irrespective of the location (Table 2). In Fort Ternan, anopheline late instars were two and a half times more likely to occur in grassy habitats (OR 2.50; P < 0.001) as well as in habitats with other vegetation cover (OR 2.40; P < 0.001) when compared to habitats without vegetation cover.

In Lunyerere, early instar larvae were six times more likely to occur in habitats with other vegetation cover (OR 6.38; P < 0.001), four times in grassy habitats (OR 4.36; P < 0.001) and three times in habitats with agricultural crops (OR 3.21; P < 0.001) when compared to habitats with no vegetation. Late instar larvae were, however, five times more likely to be sampled in grassy habitats (OR 5.44; P < 0.001) and almost four times more likely to occur in habitats covered with agricultural crops (OR 3.86; P < 0.001).

In Nyalenda, the vegetation type within larval habitats was important only for early instars (all with P ≤ 0.001) but not for late instars (all with P > 0.05) compared to habitats without vegetation (Table 2). Early instar larvae were twice as likely to be sampled in habitats covered with algae (OR 2.06; P < 0.001), grass (OR 2.08; P < 0.001) and papyrus reeds (OR 2.50; P < 0.001) compared to habitats without vegetation cover.

### Dynamics of *Anopheles *larvae in temporary and permanent habitats

Both early and late instar larvae were more abundant in temporary habitats than in permanent habitats in Fort Ternan, whereas in Lunyerere and Nyalenda the abundance was slightly higher in permanent habitats (Table 3). Temporary habitats were transient in nature and more common during the rainy season when compared to the permanent habitats. In Fort Ternan, the abundance of early instars in permanent habitats was 40% less (OR 0.586 P < 0.001) and that of late instars was 60% less (OR 0.499 P < 0.001) compared to temporary habitats (Table 3).

**Table 3:** The abundance of early and late anopheline larvae in temporary and permanent habitats per site during the two years of weekly sampling

Site	Habitat stability	No. of times sampled	Early instars (EMM ± SE)	P	Late instars (EMM ± SE)	P
Fort Ternan	Temporary	984	0.442 ± 0.029		0.347 ± 0.028	
	Permanent	811	0.243 ± 0.031	P < 0.001	0.151 ± 0.029	P < 0.001

Lunyerere	Temporary	977	0.296 ± 0.049		0.220 ± 0.048	
	Permanent	997	0.395 ± 0.048	P < 0.001	0.296 ± 0.048	P = 0.001

Nyalenda	Temporary	961	0.535 ± 0.020		0.249 ± 0.019	
	Permanent	979	0.578 ± 0.023	P = 0.027	0.334 ± 0.022	P < 0.001

OR = Odds ratio; P = Level of significance; EMM = estimated marginal means; SE = standard error of the mean

**Figure 1:**
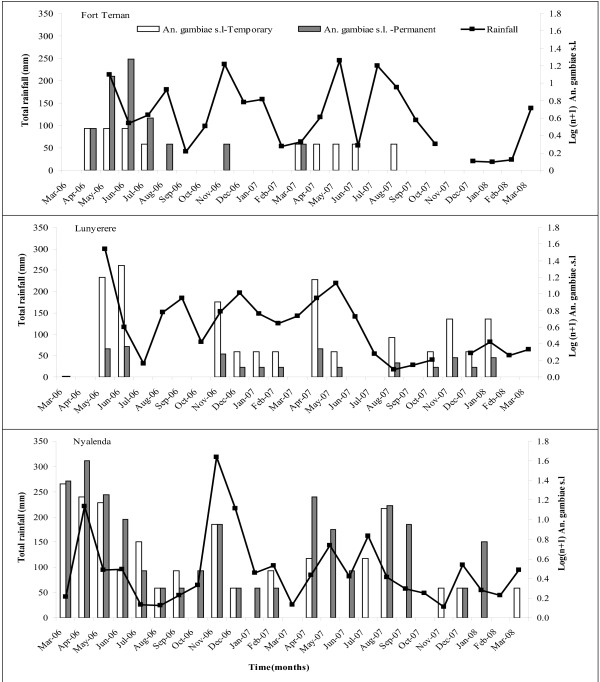
**Dynamics of Anopheles larvae**. Monthly dynamics of *An. gambiae *s.l. larvae in temporary and permanent habitats in western Kenya (Fort Ternan, Lunyerere and Nyalenda) from March 2006 to March 2008.

*Anopheles gambiae *s.l. larvae exhibited seasonal and temporal differences as shown in Figure 1. In Fort Ternan, initially *An. gambiae *s.l. was recorded more from permanent habitats and somewhat later in time in temporary habitats. The months that received low to almost no rainfall such as from September 2007 to March 2008 saw the majority of the larval habitats dry up with the exception of leaking taps. In Lunyerere, although *An. gambiae *s.l. was recorded more often from temporary habitats, whenever present, it was also recorded in the permanent habitats. The highest densities of *An. gambiae *s.l. were recorded from Nyalenda with no difference in timing of appearance of first instar larvae between temporary and permanent habitats.

### Meteorological variables and Anopheles larval abundance

**Figure 2:**
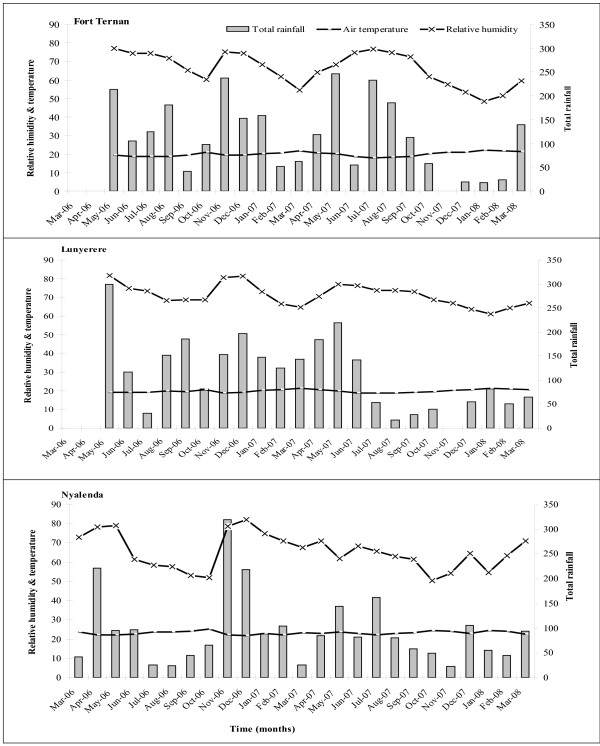
**Meteorological data**. Monthly air temperature, average relative humidity and rainfall in Fort Ternan, Lunyerere and Nyalenda from March 2006 to March 2008.

Figure 2 shows the monthly variations of average temperature, relative humidity and the total rainfall recorded during the study period. The mean monthly air temperature did not show much variation within each of the three sites (Figure 2). The peaks in relative humidity coincided with higher amounts of rainfall and led to increases in larvae in the following month. The year 2006 was an El Niño year with high rainfall in November and December. The highest rainfall was recorded in Nyalenda (Figure 2). Overall, Nyalenda had a significantly higher average temperature (22.75°C) with slightly less rainfall compared to Fort Ternan and Lunyerere, which were relatively similar in average air temperature (19.97 and 19.88°C) with high rainfall (Table 4). Analysis of variance showed significant site differences in temperature (P < 0.001) and relative humidity (P < 0.01) while total rainfall did not show any significant differences among the sites (P > 0.05). Fort Ternan and Lunyerere had average weekly temperatures >19°C and an average maximum relative humidity of >80% providing conducive conditions for malaria transmission. Nyalenda provided more favourable conditions for malaria transmission with average temperatures >22°C and a maximum relative humidity of 93%.

**Table 4:** The mean values and 95% confidence intervals of monthly minimum, maximum and average temperature, relative humidity, and total rainfall within the study sites

Variable	Statistic	Fort Ternan N = 97	Lunyerere N = 100	Nyalenda N = 52	F_(2,249)_	P
Average temperature	EM ± SE	19.97 ± 0.12	19.88 ± 0.12	22.75 ± 0.17	115.51	< 0.001*
	95% C.I	19.73-20.21	19.64-20.11	22.42-23.07		
Average RH	EM ± SE	66.06 ± 0.98	71.33 ± 0.96	71.73 ± 1.33	9.36	< 0.001*
	95% C.I	64.14-67.99	69.44-73.23	69.20-74.35		
Average RH (min)	EM ± SE	39.40 ± 1.2	47.54 ± 1.10	44. 32 ± 1.53	13.51	< 0.001*
	95% C.I	37.20-41.61	45.37-49.72	41.31-47.34		
Average RH (max)	EM ± SE	86.50 ± 2.76	90.30 ± 2.71	100.80 ± 3.76	4.76	>0.001*
	95% C.I	81.07-91.92	84.96-95.65	93.39-108.21		
Rainfall	Average	114.26 ± 15.21	124.76 ± 17.03	93.80 ± 14.30	0.34	>0.05
	95% C.I	21.13-33.21	18.57-30.47	20.20-36.70		

* Significantly different among the sites; RH = relative humidity; EMM = estimated marginal means; SE standard error; C.I = confidence interval; P = Level of significance

### Rainfall and *Anopheles *larval abundance

In Fort Ternan cross correlation analysis showed that with no time lag, weekly rainfall significantly led to high densities of early instars in both temporary and permanent habitats. With a two-week time lag early instars increased in densities with increase in rainfall while late instars increased with a three-week time lag in both permanent and temporary habitats. Weekly larval abundance was not significantly affected by weekly rainfall intensity in Fort Ternan (all with P >0.05). However, early instars were two times (OR 2.108 P >0.05) more abundant during heavy rains when compared to periods without rain (Table 5). By contrast, late instars were reduced by 19% (OR 0.809 P > 0.05) when rainfall was low.

**Table 5:** The abundance of weekly early and late anopheline larvae with rainfall intensity in Fort Ternan, Lunyerere and Nyalenda during the two years of weekly sampling

Site	Weekly rainfall (mm)	Frequency (%)	Early instars	P	Late instars	P
			Odds ratio		Odds ratio	
Fort Ternan	0	2.2	1		1	
	1-50	15.4	1.247	0.580	0.809	0.521
	51-100	72.5	1.382	0.463	1.089	0.819
	101-200	9.9	2.108	0.169	1.112	0.870

Lunyerere	0	8.5	1		1	
	1-50	75.5	1.166	0.756	1.066	0.901
	51-100	13.8	1.621	0.360	1.048	0.939
	101-200	2.1	0.682	0.795	0.982	0.987

Nyalenda	0	10.9	1		1	
	1-50	78.3	0.676	0.869	0.426	0.001*
	51-100	9.8	0.301	0.431	0.145	0.248
	101-200	1.1	0.712	0.429	0.061	0.788

In Lunyerere cross-correlations of rainfall and early instar densities showed a positive correlation (P > 0.05) at a time lag of three and five weeks while late instars showed a positive correlation at a five-week time lag. Similar to Fort Ternan, the weekly rainfall intensity had no significant effects on both early and late instar abundance (all with P > 0.05). However, heavy rains reduced early instar abundance by 32% (OR 0.682 P > 0.05) when compared to periods without rain (Table 5).

In Nyalenda, cross-correlations with rainfall showed that in temporary habitats, at time lag zero, increase in rainfall led to significant (P < 0.05) decrease in abundance for late instars while the abundance of larvae in permanent habitats did not show any relationship with rainfall. A low weekly rainfall intensity caused a significant (P = 0.001) effect on weekly abundance of late instars. Overall, increase in weekly rainfall intensity led to subsequent reduction in larval abundance (Table 5).

### Anopheles species composition

Of all *An. gambiae *sensu lato collected in Fort Ternan, Lunyerere and Nyalenda and analysed by PCR, 29, 93 and 7%, respectively, were *An. gambiae s.s*. and 71, 7 and 93% were *An. arabiensis. Anopheles arabiensis *was the main malaria vector species in Nyalenda and Fort Ternan while *An. gambiae s.s*. was the most abundant anopheline species in Lunyerere. *Anopheles funestus *was present only in the highland villages but much more abundant in Lunyerere as opposed to Fort Ternan, where only a few individuals were found (Table 6). *Anopheles coustani *was present in all sites, while *Anopheles christyi *was only found in Fort Ternan. Other anophelines collected include varying proportions of *Anopheles marshalli, Anopheles garnhami, Anopheles implexus *and *Anopheles squamosus*.

**Table 6:** *Anopheles *species composition identified from a subsample of late anopheline instars collected during sampling in the three study sites

Species	Number of mosquitoes collected		
	
	Fort Ternan	Lunyerere	Nyalenda
*Anopheles gambiae sensu stricto*	12	289	18
*Anopheles arabiensis*	29	21	231
*Anopheles funestus*	4	154	0
*Anopheles coustani*	83	29	46
*Anopheles christyi*	18	0	0
Other non vector anophelines	183	79	90

## Discussion

This study has revealed that drainage canals, hoof prints, tire tracks, rice paddies and watering taps, either leaking or broken with a constant flow of water, were important mosquito breeding habitats. These habitats were either man-made or associated with human activities. By contrast, natural water bodies such as erosion pits and habitats along the river fringe, were poorly represented among the available mosquito habitats, even in the rainy season. In summary, with availability of water (rainfall), our results indicate that anopheline larval abundance varied depending on the habitat suitability, habitat stability, vegetation covering the habitat and rainfall intensity.

Natural water sources such as pools and erosion pits relied on rainfall for water supply and in the absence of rain the habitats dried out and hence ceased to contribute to the local vector population. In contrast, habitats along the river fringe were more important for breeding during the dry season when the water levels reduced, stagnant pools of water suitable for mosquito breeding were created. In addition, when it rained heavily, flooding would occasionally occur especially in habitats along the river fringe, erosion pits and within drainage canals rendering the habitats unsuitable for mosquito breeding. This was facilitated more by the topography of the area. For Fort Ternan due to the V shaped valleys water drained into Kipchorian river down hill. In contrast, Lunyerere with its basin shaped valleys all the drainage canals were flooded while for Nyalenda being more less flat, the whole area flooded.

Results in Fort Ternan and Nyalenda show that temporary habitats were more preferred breeding habitats for both anopheline and culicine (data not included) mosquitoes. Conversely, in Lunyerere anopheline larvae occupied temporary and permanent habitats equally. These results are similar to reports by Fillinger *et al *[15] who found that semi-permanent and permanent habitats were suitable for proliferation of both anophelines and culicines. Temporary habitats due to their transient nature they held water for a shorter period of time whereas permanent habitats held water for a long period of time and after the rains, these habitats were more preferred by the culicines as observed in Fort Ternan and Nyalenda (S.S.I., personal observation). Of the highland villages studied, though at similar altitudes, more vector species were recorded from Lunyerere and in higher abundance than in Fort Ternan. In Lunyerere the mosquito breeding habitats were mainly in the broad, flat, valley floor, which until recently was a natural swamp forest. Lunyerere experienced underground seepage of water that ensured the presence of abundant, clean sunlit shallow bodies of water that were preferred mosquito breeding habitats all year round. By contrast, the Fort Ternan valley is narrow with steep slopes, causing a rapid drainage of water unless human activities create or construct water-retaining structures. In addition, Paaijmans *et al *[28] showed that the mean daily water temperatures (in small, medium and large pools) in these sites were generally higher than the mean air temperature, which eventually led to rapid development times. Nyalenda was a natural drainage area, which in the last decade has been turned into an agricultural area linked to the urban growth of Kisumu city, creating many variable water bodies suitable for mosquito breeding.

This study revealed that habitats that had grass growing in them had more anopheline mosquitoes than habitats with other vegetation types and open habitats. A previous study by Fillinger and others [15] found similar results. These findings were apparent in the two highland village sites and suggest that grass protected mosquito larvae from being swept or flushed away by running water [29] and/or predation [30,31]. Grass could have also been convenient in offering newly emerged adult and gravid mosquitoes a shaded resting site. The role of grass as a resting site was not investigated and more research is needed on this topic. Habitats containing papyrus reeds were more important for anopheline larval breeding in Nyalenda. Natural and undisturbed papyrus swamps were found unsuitable for anopheline breeding [32,21]. However, once the natural state of *Papyrus *spp. is disturbed, as was the case in Nyalenda and Lunyerere, mosquito breeding can occur due to incursion of other vegetation. Consequently, this may lead to an increase in mosquito-human contact, eventually leading to an increase in malaria transmission.

*Anopheles arabiensis, An. gambiae *and *An. coustani *were present in all three study sites. *Anopheles arabiensis *comprised 71% of *An. gambiae *s.l. collected from Fort Ternan. This is the highest proportion of this species that has ever been recorded in the highlands of western Kenya. Previous studies in the highlands of western Kenya report the presence of *An. gambiae*, but not *An. arabiensis*, a major vector species present in the Lake Victoria basin [33-35]. The presence of *An. arabiensis *in Fort Ternan and Lunyerere can be attributed to the slow changes in land use, such as deforestation that lead to changes in micro-climatic conditions favouring the survival of this species. The air temperatures used in the present study may have been lower than the corresponding water temperatures in the sampled habitats, a factor that seems to favour the existence of *An. arabiensis *in high altitude areas. In their study Paaijmans *et al *[28], have shown clearly that the use of air temperature rather than water temperature will tend to underestimate the population growth rates of mosquito populations. *Anopheles funestus *was only found in the highland villages, while *An. christyi *was only present in Fort Ternan, in line with previous studies by Koenraadt and others [35]. The vector status of *An. christyi *has not been resolved, although Garnham [36] suggested that the species might occasionally be implicated as a malaria vector. *Anopheles coustani *was present in all the study sites and this species has recently been reported as a possible malaria vector in East Africa [37]. In Nyalenda, a site where on many occasions the water was polluted with human waste and other debris (S.S.I, personal observation), *An. arabiensis *was more abundant than any other anopheline species. *Anopheles *species seem to have adapted to breeding in environments rich in organic waste, which was also noted in urban Dar es Salaam [10,11] contrary to the findings of Robert *et al *[38]. *Anopheles gambiae *was not found in Nyalenda, which is in line with recent changes of the distribution of this species in the Lake Victoria basin, where it seems to be replaced by *An. arabiensis*, especially in agricultural areas [31].

These results indicate that whenever it rained in Nyalenda, anopheline larvae were reduced with heavy rains leading to extreme reductions in larval abundance. It is possible that heavy rains flushed away and/or killed the larvae. These results are supported by experiments by Paaijmans *et al *[29], who reported that precipitation flushed, ejected and killed *An. gambiae *larvae in a semi-natural environment. Contrary to these results, in Fort Ternan when it rained heavily, more young larvae were recorded with a time lag of fourteen days, while late larvae were recorded in twenty-one days. At a slightly longer development period when compared to Nyalenda, which had no time lag between rain and the presence of larvae. In these highlands, the rains seem to have provided new habitats suitable for oviposition and larval development.

The current study was done under natural conditions and external factors could have played an important role in the colonization and growth of mosquito larvae in the respective habitats. Factors such as water turbidity, nutrient content of the water, cannibalism, predation of immature stages, parasitism, pathogens, competition, water temperature and plant odours that could have either repelled or attracted female mosquitoes during oviposition [39-45], were not controlled, which could have played a role in the results obtained. Studies on the ecology of larval anopheline mosquitoes are methodologically challenging and the current study had several limitations. Quantitative sampling was problematic because the standard dipping technique adapted for this study was unsuitable in small habitats and consequently the density of mosquito larvae and pupae may have been underestimated [25,46]. In this study, productivity of a habitat is expressed as the density/abundance of larvae [5,6,39-41] and not as the number of pupae or adult mosquitoes that emerged from a habitat [46-48]. Although larvae were recorded in many habitats, not all larvae might have successfully developed into adults. However, because spatial models have shown the importance of non-productive habitats as part of transmission foci in facilitation of mosquito movement in search of a blood meal and aquatic habitats [49,50], it is inevitable to focus anopheline larval control both in productive habitats and non-productive habitats.

The study has shown that mosquito larval abundance depends on the suitability, stability and productivity of the larval habitat and rainfall intensity. Suitability of any given habitat may be influenced by the vegetation type within the larval habitat and the land use in the area under study. Most of the mosquito larval habitats in the studied areas were a result of human activities. Lunyerere (highland) and Nyalenda (western lowlands) represent reclaimed swampy areas turned into farmland. This land use change seems to have contributed significantly to the suitability of habitats for anopheline mosquito breeding. By contrast, in Fort Ternan (highland), mosquito breeding was found not to be directly related to crop farming but rather to livestock holding, as most anopheline larvae were collected from cattle hoof prints. With the increasing human population in the highlands, enhanced human activities including deforestation, farming and livestock rearing are likely to create more vector habitats [15,17,18,30,46,51].

This was a baseline study that provided insights in the development of integrated larval source management strategies suitable for the larval habitats present in the respective study sites. An integrated larval source management approach such as water and environmental management measures, biological control options like predatory fish/larvae are highly feasible [52], in addition to the use of microbial larvicides, especially in temporary habitats and in areas where water cannot be drained away. Considering the current study sites, source reduction through drainage and habitat manipulation would be suitable for Lunyerere and Fort Ternan. Whereas the application of microbial bio-larvicides such as *Bacillus thuringiensis *var *israelensis *(*Bti*) would be a better option for Nyalenda, because the community relies on the same water for domestic purposes [53]. Due to the transient nature of temporary habitats, microbial bio-larvicides are most suitable irrespective of the area under study [54]. Such mosquito vector control programmes should work closely with land and home-owners, as the majority of the mosquito breeding habitats were man made.

## Competing interests

The authors declare that they have no competing interests.

## Authors' contributions

The study was conceived by SSI and WT. SSI supervised field data collection, analysed the data and drafted the manuscript. KPP assisted with data analysis and together with WT in writing of manuscript. AKG, WRM, RL and WT assisted with study design and logistical issues. All authors read and approved the final version of manuscript.
